# Preventive medicine as a first- or second-choice course: a cross-sectional survey into students’ motivational differences and implications for information provision

**DOI:** 10.1186/s13104-017-2706-6

**Published:** 2017-08-10

**Authors:** Van Anh Thi Nguyen, Karen D. Könings, Albert J. J. A. Scherpbier, Pamela Wright, Hoat Ngoc Luu, Jeroen J. G. van Merriënboer

**Affiliations:** 1 Department of Medical Education and Skills Laboratory, Hanoi Medical University, 1 Tonthattung, Dongda, Hanoi, Vietnam; 2 School of Health Professions Education (SHE), Faculty of Health, Medicine and Life Sciences, Maastricht University, P.O. Box 616, 6200 MD Maastricht, The Netherlands; 3 General Director of Medical Committee Netherlands Vietnam, Weteringschans 32, 1017 SH Amsterdam, The Netherlands; 4 Biostatistics and Medical Informatics Department, Institute of Training for Preventive Medicine and Public Health, Hanoi Medical University, 1 Tongthattung, Dongda, Hanoi, Vietnam

**Keywords:** Career choice, Motivation, Preventive medicine, First choice, Second choice

## Abstract

**Background:**

Challenges in recruiting and retaining medical staff in preventive medical specialties have recently been the subject of numerous studies. To improve selection procedures, it is important to understand the career preferences and incentives of students in preventive medicine (PM), who initially marked the program as either their first choice or second choice. 1386 PM students in four Vietnamese medical schools participated in a survey using a structured, written questionnaire. Students were asked about their reasons for entering medical school and studying PM, their perceptions of PM during the academic course, and their expected career path following graduation.

**Results:**

First-choice PM students (group 1) more often had siblings working as a preventive doctor, while second-choice PM students’ siblings (group 2) were more often medical students or clinical doctors. Group 1 had gathered more information about PM by consulting their high-school teachers and the national career guide. They were mainly drawn to the PM program by the newness of the profession, the prospect of a high-income job, its low entry criteria and low study burden compared to general medicine, their desire to uphold their family tradition, and to fulfill their family’s wish of having a doctor in the family. Group 2 chose to study PM because they wanted to pursue their dream of becoming a doctor. Compared to the first group, their perception of PM more frequently changed during the later years of the curriculum and they more frequently envisioned becoming a clinical doctor following graduation.

**Conclusions:**

Interest in and motivation for PM may be cultivated among prospective or current students by improving information provision, diffusing knowledge, and otherwise acquainting students better with the PM specialty before and during the program.

**Electronic supplementary material:**

The online version of this article (doi:10.1186/s13104-017-2706-6) contains supplementary material, which is available to authorized users.

## Background

Despite the important role primary health care (PHC) plays in health care systems, in many countries the career of a primary health worker is not held in very high regard. Consequently, few medical graduates choose to work in this field. In comparison with clinical specialties of “high prestige,” PHC specialties, such as rural health care, occupational health, public health, and preventive medicine (PM), are considered problematic areas when it comes to the recruitment of students and retention of graduates to work in this field [1, 2]. According to the 2009 annual report of the Ministry of Health in Vietnam [3], the majority of its university pharmacists (82%), doctors (59%), and nurses (55%) work in urban areas, while the population in these areas accounts for only 27% of the total population. A recent study on the general perception about PHC and career choice at PHC settings among 400 final year medical students in Vietnam revealed that, although almost all students (99%) agreed that PHC is very important, less than 1% of them intended to work in primary care settings (i.e. communal and district health facilities) and only 3.7% of them intended to work as a preventive medical doctor [4].

Other works have proved that medical students’ career preferences are influenced by biographical characteristics such as gender, by having a physician in the family, and urban or rural background [4–9]. Female students show a higher preference for PHC specialties, such as community medicine and family medicine, than their male counterparts [5, 6]. It is also reported that students from rural areas (i.e. students of rural origin, grown up in a rural area, or having family who live in a rural area) are more likely to enter rural practice than those from urban areas [7]. Although medical students who have a physician in their family show a higher proclivity to choose non-primary care specialties than other students [5, 6], information about this factor in students who choose PHC specialties is absent. Studies have shown a trend toward smaller numbers of students whose top choice is preventive specialties [4, 5, 8, 9]. Plausible explanations for this development include: lack of specialty information [4], equal appeal of several other specialties [5], desire for monetary rewards [8], and lack of interest due to little or negative appreciation of the specialty’s attributes [9]. The lack of ambition to pursue a career in preventive care is not only manifest among freshmen but also among graduates. Students usually become more changeable in their choice of PHC specialties in the clinical phase or senior year, which may be explained by students’ exposure to clinical work and desire to have a high-prestige career [1].

Vietnam has created a separate curriculum leading to an undergraduate degree in PM with the aim of providing specialized PM medical staff who will work in preventive medical centers and in the community. Bachelor and Master degrees in public health provide non-medical staff for management of programs which are often PM programs. Concerns have been voiced, however, that medical graduates who have completed the PM curriculum will eventually not opt for a career in PM, but seek a clinical position after taking postgraduate clinical specialty training. That is not the intention but does happen, according to observations by senior educators and teachers in PM and public health education in Vietnam [10], though more evidence is needed to confirm the observations. Additionally, society seems to hold a prejudice against the PM profession, deeming it secondary in prestige to any clinical/hospital profession. In the study among 400 final year medical students, researchers have found that students recognized the low status of PHC work in Vietnam and did not consider that working in PHC would contribute to their professional advancement [4]. Another study in two provinces in Northern Vietnam found that health workers’ willingness to work in rural areas was being compromised by the following factors: low incomes, bad working conditions, and a lack of appreciation of the preventive specialties from the community at large, partly because of their relative unfamiliarity with the profession compared to curative professions [11]. These issues were confirmed by results of another study on job motivation of rural health workers in Vietnam, which listed five main factors discouraging them in their work, including: low income and allowance, difficult transportation, no prospect of continuing development, and heavy work load without clear plans [2].

Further efforts to increase the number of physicians serving in preventive specialties and rural areas have abounded, involving high-school students, medical students, and doctors working in primary health care sectors. Strategies such as adapting student selection and admission procedures, or early exposure of students to training in rural sites have also been adopted to increase rural students’ participation and motivation [12, 13]. However, these studies focused on medical students and their motivation to work in preventive specialties after graduation. In the Vietnam situation, we could focus on students who had already made a choice to study PM from the beginning. It would be important to find out what kind of students are attracted to a study in PM and for what reasons, what their perceptions are during the course of the program, and what they expect from their future job. This information might provide a scaffold for career guidance, selection and training activities of the medical school to improve the quantity and quality of PM graduates.

To develop a suitable and effective curriculum that encourages students to study PM and to pursue it as their future profession, it is necessary to elucidate the differences between the students who made a primary choice for PM (group 1) and those for whom it was the second choice (group 2). This study, therefore, investigates the differences between these two student groups with regard to: (1) their personal characteristics; (2) the sources of information about PM they accessed before choosing the specialty; (3) their reasons for entering medical school and studying PM; (4) their perceptions of PM and constancy of that opinion throughout the course; and (5) their expected career path following graduation. The outcome will provide a baseline for further efforts at making sound policy recommendations on how students’ motivation to study and work in PM might be increased.

## Methods

### Participants and setting

This cross-sectional survey was conducted in four medical universities located in Northern Vietnam. At these universities, students are trained to work in different areas of medicine, such as PM, general medicine, traditional medicine, public health, and nursing. To be enrolled in a medical university, each candidate applies for a studentship position in two different areas: their first choice and second choice. Based on the results of a national entry examination, universities consider and select students for the training in either their first or second choice. Candidates who do not get accepted into their preferred program can still be admitted to the second choice area, as long as their exam results satisfy the enrollment criteria for that one.

All 1404 PM students from the first to the sixth year in the four medical universities were invited to participate voluntarily in this study. The rate of participation was very high (1386 respondents, 98.7%), probably because students were invited to participate immediately after attending lectures, while still in the lecture hall, and were given a small financial compensation (2 euros/student) for their time (approximately 20 min) on answering the questionnaire. Of these respondents, 936 (67.5%) were students for whom PM was their first choice, and 450 (32.5%) were students whose first choice was general medicine (82.78%), dentistry (9.09%), or other courses (8.14%). Hereinafter we will refer to these groups as “first-choice students” and “second-choice students”. The mean age of the respondents was 21.57 years (*SD* = 2.24), and 61% were female.

### Materials

The questionnaire was designed based on existing questionnaires of previous studies on career preference in medicine [2, 7, 10] as well as on the results of group discussions with students from PM and general doctors’ training courses to create a suitable version for the Vietnamese situation. A pilot study was performed with 12 PM students in Hanoi from different course years to improve the format and the clarity of the items. The final version used incorporated the results from the pilot study (Additional file 1).

The questionnaire comprised a series of 33 questions in three parts. The first part included 15 items on basic personal socio-demographic data and parents’ education and profession. These items also asked whether students received any assurance or any other type of assistance from family, relatives, or friends, and whether they expect help to find a job in the health care sector after graduation. The second part contained 15 items about whether PM was their first or second choice when applying to the medical university, reasons for choosing to study PM, who had the biggest influence on their decision to study PM, and their perceptions of PM over time. The last part consisted of three items about their expectations of future jobs. Participants were asked to tick those predefined options that best fitted their opinion; for some of the items, they were allowed to select more than one option (but maximally three). In each question, there was an option to respond “Other” followed by space to clarify, to give an opportunity for participants to give unrestricted responses.

### Data analysis

We conducted a descriptive analysis of the answers. Variability according to preference for PM (first/second choice) was analyzed by comparing the different strata with χ^2^ test. Due to the multiplicity of tests, the Bonferroni correction was applied to control for the risk of inflation of type I errors. Results were considered statistically significant if the two-tailed *p* value was less than .01. Subsequently, a post hoc analysis on standardized residuals was used to report exactly which differences were at a level of significance. Data were analyzed using IBM SPSS Statistics (version 20.0).

## Results

### Students’ personal characteristics

Between first-choice and second-choice students, no statistically significant differences were found in gender, age, or other socio-demographic characteristics such as rural background, parents’ education, and parents’ medical-related jobs. The only significant differences between them were their year of study and having a sibling with a health-related job (see Table 1). In the higher years, there were more second-choice students, while in the basic years (i.e. the more recently recruited) the number of first-choice students was highest. First-choice students more often had siblings working as preventive doctors; conversely, second-choice students more often had siblings who were medical students or clinical doctors. Of all students, 36.51% (38.39% first-choice students, 31.25% second-choice students, *χ* ^*2*^ = 3.09, *p* > .05) said that they were assured of a job in medicine after graduation, aided either by family members or by relatives and friends.

**Table 1:** Comparison between two groups, regarding study level and having sibling working in health related job

	First-choice students			Second-choice students				
	n	%	Std. R	n	%	Std. R	χ^2^	p
	N = 936			N = 450				
Level of study								
Basic years	396	43.21	3.87	87	19.33	−5.58	98.49	.000*
Clinical years	270	28.85	.41	120	26.67	−.59		
PM years	270	28.85	−4.11	243	54	5.92		

	N = 298			N = 142				
Sibling with a health related job								
Student in medicine	64	21.48	−1.07	44	30.99	1.55	11.65	.009*
Clinical doctor	38	12.75	−.91	27	19.01	1.31		
Preventive doctor	10	3.36	.93	1	.70	−1.35		
Other medical jobs	186	62.42	.96	70	49.30	−1.39		

* Significant difference between first-choice and second-choice students (with Bonferroni adjustment)

### Obtaining information about PM

First-choice students had made more efforts to obtain information from many sources about PM before deciding to choose the specialty than had second-choice students (58.30% vs. 43.80%, respectively, *χ* ^*2*^ = 24.95, *p* < .01). Among information sources as the media, the national career guide, parents, relatives, high school teachers and friends, students most frequently turned to the media for information about PM, including the Internet, television, and newspapers. However, consultation of two information sources differed significantly between the two groups: high-school teachers (*χ* ^*2*^ = 6.94, *p* < .01) and the national career guide with information about future professions (*χ* ^*2*^ = 35.22, *p* < .01) were more often used by first-choice students than by second-choice students.

### Reasons for entering medical school and studying PM

**Fig. 1:**
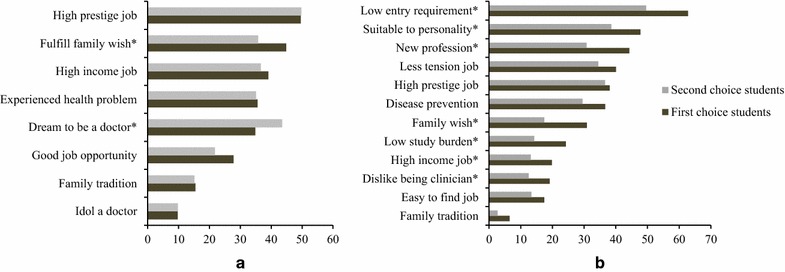
Reasons for students to enter medical school (**a**) and study PM (**b**). *Asterisks* significant difference between first-choice and second-choice students (with Bonferroni adjustment)

The results on the left in Fig. 1 show the significant difference in the reason that the two groups of students were drawn to medical school. While first-choice students were primarily drawn to medical school by the desire to fulfill their family’s wish (*χ* ^*2*^ = 10.32, *p* < .01), for second-choice students their own dream of becoming a doctor proved more decisive (*χ* ^*2*^ = 9.86, *p* < .01).

The results on the right in Fig. 1 represent the reasons for studying PM. Significantly more often than did second-choice students, first-choice students opted for a study in PM because of the benefits the future profession would bring, being relatively new (*χ* ^*2*^ = 22.93, *p* < .01) with high-income prospects (*χ* ^*2*^ = 9.34, *p* < .01). First-choice students were also more drawn to the program than were second-choice students because of not wanting to become a clinical doctor (*χ* ^*2*^ = 9.43, *p* < .01), the low entry requirements (*χ* ^*2*^ = 81.83, *p* < .01), and the low study burden (*χ* ^*2*^ = 18.12, *p* < .01) specific to PM training. In addition, more than second-choice students, first-choice students held the opinion that pursuing a career in PM suited their personality *(χ* ^*2*^ = 10.24, *p* < .01), and that it would also help them to fulfill their family’s wish (*χ* ^*2*^ = 28.19, *p* < .01) and uphold their family tradition (*χ* ^*2*^ = 8.94, *p* < .01).

### Perceptions of PM students

In the program of their study, second-choice students changed their attitude toward PM more frequently than did first-choice students (57.82% vs. 41.08%; *χ* ^*2*^ = 33.65, *p* < .01). Of the students who changed their attitude toward PM, 72.7% of first-choice students and 77.25% of second-choice students gained a better appreciation of PM (*χ* ^*2*^ = 1.65, *p* > .05). At the time of the survey, however, 40.58% of the second-choice students still regretted their choice to study PM and wished to change to another profession, while only 28.41% of the first-choice students reported similar feelings (*χ* ^*2*^ = 22.65, *p* < .01). Another striking difference was the fact that 55.26% of first-choice students changed their attitude during the pre-clinical years (years 1–2), while 58.36% of the second-choice students only did so during the clinical years (years 3–4) and PM years (years 5–6) (*χ* ^*2*^ = 20.31, *p* < .01).

**Fig. 2:**
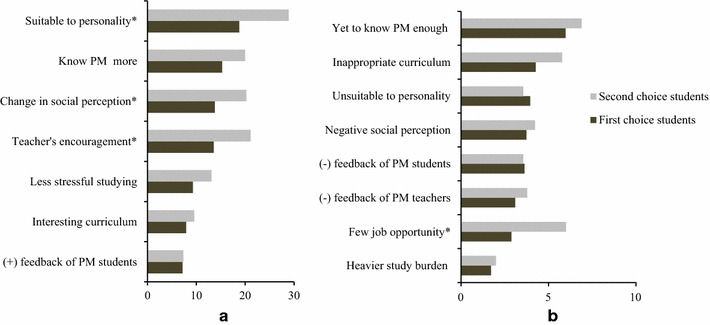
Reasons making students appreciate PM more (**a**) and less (**b**). *Asterisks* significant difference between first choice and second choice students (with Bonferroni adjustment); (+) feedback: positive feedback, (−) feedback: negative feedback

Figure 2 presents the reasons for this change in attitude. It indicates why more second-choice students—as opposed to first-choice students—changed toward a better appreciation of PM: they gained a more positive social perception of the PM profession (*χ* ^*2*^ = 9.44, *p* < .01), they were encouraged by PM teachers (*χ* ^*2*^ = 12.85, *p* < .01), and they realized that PM suited their own personality better than initially expected (*χ* ^*2*^ = 17.97, *p* < .01). The main reason for lower appreciation of PM by second-choice students was the prospect of fewer job opportunities (*χ* ^*2*^ = 7.88, *p* < .01).

### Projected career path following graduation

More second-choice students than first-choice students anticipated landing jobs as clinical doctors in hospitals (*χ* ^*2*^ = 42.17, *p* < .01). Conversely, more first-choice students than second-choice students anticipated choosing a job within the PM specialty (*χ* ^*2*^ = 7.76, *p* < .01), with second-choice students being more interested in jobs that offer the opportunity to continue their studies (*χ* ^*2*^ = 8.06, *p* < .01) and that are located at provincial health care centers (*χ* ^*2*^ = 9.31, *p* < .01).

## Discussion

This study reveals the differences between students in PM who, prior to enrolment, selected the program as their first choice and those who did not. We found that in the higher study years there were a higher proportion of second-choice students, while the earlier years, that is, the later intake, had a higher proportion of students for whom PM was the first study choice. This reflects a trend of students increasingly selecting a study in PM at medical school as their first preference, which bodes well for the recruitment of students in PM. Previous studies on career choice have pointed that medical students who have a physician in the family are more likely than other students to choose non-primary care specialties [5, 9]. In our study, although there were no statistically significant differences between the two groups in gender, rural background, parents’ education, and parents’ medical-related jobs, those students for whom PM was the first choice had more often a sibling working in PM and non-clinical specialties than did second-choice students. This finding reinforces the impact of the “medical family” factor, albeit viewed from the reverse perspective of primary care specialties.

The fact that barely half of the second-choice students collected information about PM before selecting the specialty makes sense, as PM was not their first preference. At the same time, however, the number of first-choice students obtaining information was also surprisingly low—only 58.30%—while one would expect this to be much higher. The finding that more first-choice students had family already working in PM could explain partly for this low number, because they probably know things already without actively searching. However, the overall results suggest that students make their choices without being sufficiently informed about the specialty. A lack of information also arose as one of the factors influencing the tendency to prefer preventive specialties over other specialties [4, 9]. Another finding was that students frequently appealed to the media and national career guide for information. However, these sources only provide general and unsystematic information about, for example, the job of a PM doctor, the names of medical schools offering PM training, the number of training places per year, and so forth. In our study, that so many PM students selected general doctor as their first choice at the beginning (82.78%) also reflects the public understanding which is more familiar from the media with the function of the general doctor. This information gap could be bridged by improving the quality of career guidance services to cultivate interest in the health professions among high-school students [14] and by providing the media with more examples of the good work done by PM doctors. In our study, first-choice students frequently turned to their high-school teachers or the national career guide for information about PM, underlining the important role such information sources play for students.

It is not surprising that about 40% of second-choice students regretted their choice and wanted to change profession, at the time of the survey, while close to one-third of first-choice students reported similar feelings. More than half of the first-choice students changed their views (toward more or less appreciation of PM) already in the first 2 years of the program, while nearly 60% of second-choice students had shifted their grounds in the later years, by the time they did their clinical rotations and had acquired more experience and knowledge of PM. Research on career choice has shown that the way in which students experience family medicine during the later years of the curriculum is a determinant of whether or not they will select this as their specialty [2, 15]. The study of Landström et al. in 2014 [16] also emphasized the association between interest in becoming a general practitioner and wanting to insert more general practice in undergraduate training of Sweden medical students. Our data do not allow us to explain this relationship in the context of PM. However, the fact that more second-choice students than first-choice students changed toward a better appreciation of PM suggests that they were insufficiently informed about the profession at the start of the program. This could explain the changeable nature of their opinion about PM.

Our study shows that the incentives to study general medicine and PM, differed in focus. In their ambition to become a PM doctor, first-choice students were largely driven by extrinsic factors, such as: PM is a new profession and high-income job, it has low entry criteria and a low study burden compared to general medicine, and the desire to uphold their family tradition and fulfill the wish of having a doctor in their family. Second-choice students, on the other hand, were drawn to a study in PM mainly by intrinsic reasons, such as “the dream to become a doctor” regardless of specialty, and the belief that a job in the PM sector suited their own personality. This distinction was, in turn, reflected in students’ projections of future jobs: more second-choice students than first-choice students wanted to pursue a career as a clinical doctor working in a hospital, or to stay in the big cities even though much of PM work is at lower levels in the health system. Furthermore, second-choice students also preferred a job which could offer them further study opportunity and at a health care center at provincial level. This finding is consistent with results from a previous study on the PHC specialty choice of 400 final year medical students in Hanoi Medical University, in which Kim et al. [4] found that only 37.3% of medical students thought they could master their professional activities better by working in PHC. Agyei-Baffour and colleagues also reported that medical students’ intrinsic motivation to study medicine (i.e. desire to help others) did not translate into willingness to work in rural areas [7]. Intrinsic motivation results in high-quality learning and creativity, so if students do lack intrinsic motivation, educators should encourage more active forms of extrinsic motivation as an educational strategy [17]. This was reflected in real situation while Landström et al. indicated in their study [18] that if students observed a personal enthusiasm from their general practitioner supervisors, it has a positive influence on their attitude toward the specialty. In the case of PM students, if educators would provide students with more information on PM work and raise awareness of the profession’s virtues during training, this could boost their motivation to study and pursue a career in PM. From our study, second-choice students changed toward a better appreciation of PM after they were encouraged by PM teachers, as well as by realizing that PM suited their own personality better than initially expected, and the more positive social perception of the PM profession when they had more experiences during the clinical years (years 3–4) and PM years (years 5–6). However, these were only our initial findings and testing this hypothesis could be an interesting new topic for future study.

A limitation of this study was that the survey was conducted only in the four Northern medical universities in Vietnam. There may be differences in demographic characteristics and public perception about the medical profession between different regions of the country, but also between countries, that could influence the career choice of PM of students. Also, the study was a cross-sectional observation which did not include information about the final destinations of the students after graduation. Further investigation in medical schools in other parts of the country and internationally, as well as a student cohort tracer study could reveal more insight in factors that impact on the decision by students to choose and to pursue PM.

## Conclusions

This study about motivation and career preferences of students in PM revealed differences between students who had PM as their first study preference and those who only selected it as their second choice. These two groups of students differed in the way they retrieved information about the profession, the stability of their opinion about PM, and their motivation to study and pursue a career in PM. Improving information provision about the work of PM, diffusing knowledge, and otherwise acquainting students better with PM before and during the program may help to cultivate their interest and consequently to increase the number of health care staff working in the PM sector. The findings provided lessons from Vietnam which could be useful for other countries in the struggle to promote the fields of public health and preventive medicine.

## Additional file

**Additional file 1.** Survey questionnaire on career choice of Prevent Medicine students. This file contains English version of the questionnaire used in the study.

## Abbreviations

PM: preventive medicine
PHC: primary health care
