# Supplementary material for: Preventive medicine as a first- or second-choice course: a cross-sectional survey into students’ motivational differences and implications for information provision
Source: BMC Res Notes. 2017 Aug 10;10:383. doi: 10.1186/s13104-017-2706-6 (PMC5553608; doi:10.1186/s13104-017-2706-6)
Supplement: Supplementary file 1 — Additional file 1. Survey questionnaire on career choice of Prevent Medicine students. This file contains English version of the questionnaire used in the study. [file 13104_2017_2706_MOESM1_ESM.doc]

**Research participation consent form**

Hello! We are carrying out research on the motivation behind the medical student’s choice to follow the Prevent Medicine discipline at four medical schools in Vietnam. The purpose of this research is to improve the vocational guidance activity and the quality of the graduates.

The information that you provide us will be kept confidential. When the result of this research is announced, no name and personal information will be mentioned but the overall outcome. It will take you about 20 minutes to answer all the questions in the survey form. You have all the rights to refuse to answer any question, having not to be concerned about discrediting your study group.

In case you have some concerns or difficulties when participating in this research volunteer job, please contact main research officer Ms. Nguyen Van Anh via email vananh299@gmail.com

We would be thankful to your precious support.

***Would you like to take part in this research activity?***

***I have been given clear explanation about the purpose of the research, the risks and benefits to myself in taking part in the survey***

◻Yes ◻No 🡪 You can stop now

Date: ... / ... / ... . Time: .....

**Survey questionnaire on career choice of Prevent Medicine students**

| **No** | | **Question** | | **Answer** | **Answer code** | **Move to question** |
| --- | --- | --- | --- | --- | --- | --- |
| **A** | | **Personal Information** | | ***Tick into the corresponding box*** |  |  |
| A1 | What year of study are you in currently? | | | Y1  Y2  Y3  Y4  Y5  Y6 | 1🗖  2🗖  3🗖  4🗖  5🗖  6🗖 |  |
| A2 | What is your gender? | | | Male  Female | 1🗖  2 🗖 |  |
| A3 | Where is your place of birth? | | | Big cities (as Hanoi, HoChiMinh city)  Other cities  Town  Rural country side  Foreign country  Others:………………  Not know | 1 🗖  2 🗖  3 🗖  4 🗖  5 🗖  97 🗖  98 🗖 |  |
| A4 | The total length of time you have been living in rural area *(including remote highlands and lowlands)* | | | I have never lived in rural area  The number of years  Not know | 1 🗖  ……  98 🗖 |  |
| A5 | Grade your attachment to rural area, it means your feeling about the familiarities, fond remembrance when away, or family connection | | | Feel nothing  Not close  Normal  Close  Very close  Not know | 1 🗖  2 🗖  3 🗖  4 🗖  5 🗖  98 🗖 |  |
| A6 | Where are you staying for the time being?  *(Where you reside currently)* | | | At parent’s house  At your own house  Dormitory  Rental house  Hotel/hostel  Acquaintant/relative’s house  Others (in detail)…………………… | 1🗖  2🗖  3🗖  4🗖  5🗖  6🗖  97 🗖 |  |
| A7 | Where is your subsidize come from while you study at the medical school? | | | Parent/family  Scholarship  Part-time jobs  Bank loan  Others (in detail)………………… | 1🗖  2🗖  3🗖  4🗖  97🗖 |  |
| A8 | Your father’s highest level of education? | | | Post graduate  University graduate  Higher education  Technical high school  High school  Secondary school  Primary School  Illiteracy  Not know | 1🗖  2🗖  3🗖  4🗖  5🗖  6🗖  7🗖  8🗖  98 🗖 |  |
| A9 | Does your father work in health or pharmacy currently? *(not counting if your father studied medicine but works outside of health and pharmacy)* | | | No  Clinical doctor  Preventive doctor  Manager (in medical facilities)  Researcher  Medical university/college lecturer  Pharmacist  Others (in detail)………………….  Not know | 1🗖  2🗖  3🗖  4🗖  5🗖  6🗖  7🗖  97🗖  98 🗖 | 🡪 A11 |
| A10 | If in medicine/pharmacy, who does your father work for? | | | The Ministry of Health  State Hospital/research institution  Medical/Pharmacy/Public Health university/College  Provincial Health Department/hospital  District Health Department/hospital  Commune health center/infirmary  Private hospital/clinics  Pharmacy  Others (in detail)………………….  Not know | 1🗖  2🗖  3🗖  4🗖  5🗖  6🗖  7🗖  8🗖  97🗖  98 🗖 |  |
| A11 | Your mother’s highest level of education? | | | Post graduate  University graduate  Higher education  Technical high school  High school  Secondary school  Primary School  Illiteracy  Not know | 1🗖  2🗖  3🗖  4🗖  5🗖  6🗖  7🗖  8🗖  98 🗖 |  |
| A12 | Does your mother work in health or pharmacy currently? *(not counting if your mother studied medicine but works outside of health and pharmacy)* | | | No  Clinical doctor  Preventive doctor  Manager  Researcher  Medical university/college lecturer  Pharmacist  Others (in detail)………………….  Not know | 1🗖  2🗖  3🗖  4🗖  5🗖  6🗖  7🗖  97🗖  98 🗖 | 🡪 A14 |
| A13 | If in health/pharmacy, who does your mother work for? | | | The Ministry of Health  State Hospital/research institution  Medical/Pharmacy/Public Health university/College  Provincial Health Department/hospital  District Health Department/hospital  Commune health center/infirmary  Private hospital/clinics  Pharmacy  Others (in detail)………………….  Not know | 1🗖  2🗖  3🗖  4🗖  5🗖  6🗖  7🗖  8🗖  97🗖  98 🗖 |  |
| A14 | Do one of more of your brothers/sisters work in health/pharmacy? *(more than one answer possible)* | | | I don’t have any sibling  No, they don’t work in health/pharmacy  He/she doesn’t work in health sector  Student in Medicine/Pharmacy  Clinical doctor  Preventive doctor  Manager in health care sector  Medical university/college lecturer  Pharmacy  Others (in detail)………………….  Not know | 1🗖  2🗖  3🗖  4🗖  5🗖  6🗖  7🗖  8🗖  9🗖  97🗖  98 🗖 | 🡪 A15 |
| A15 | Is there any direct guarantee and influence from your family on the prospect of you getting a job in Health care sector after your graduation? | | | No, there is not  Number of family members could help you to get a job in health care sector? | 0 🗖  …… |  |
| **B** | | **Career choice** |  | |  |  |
| B1 | | What is the overall grade of your high school result? | Excellent  Good  Average  Bad  Others (in detail)…………… | | 1🗖  2🗖  3🗖  4🗖  97🗖 |  |
| B2 | | Think back when you decided to apply to join the Medical university, what were your most important reasons behind your choice? *(if more than one, then indicate their prioritizing by numbering them)* | **Characteristics of the profession**  - High moral value and humanity  - High prestige  - High income prospect  **Personal preference**  - Having an affinity for biology/ chemistry  - Just want to try if I could manage to enroll in medical university  - My childhood dream to become a medical doctor  - Family/own experience of health problem  **Influenced by others**  - One of my idol is a medical doctor  - Guaranteed job opportunity  - Family tradition in health profession  - Fulfilling family’s wish  - Following friends’ act  Others (in detail) …………  Not remember | | 1🗖  2🗖  3🗖  4🗖  5🗖  6🗖  7 🗖  8🗖  9🗖  10🗖  11🗖  12🗖  97🗖  98🗖 | ***priority***  ***…***  ***…***  ***…*** |
| B3 | | Who had the biggest influence on your decision to enroll to the medical school? *(if more than one, then indicate their prioritizing by numbering them)* | Myself  Father/mother  Sister/brother  Relatives  Friend  Partner/wife/husband  Idolized medical doctor  Others (in detail) ………….  Not remember | | 1🗖  2🗖  3🗖  4🗖  5🗖  6🗖  7🗖  97🗖  98🗖 | ***priority***  ***…***  ***…***  ***…*** |
| B4 | | Which was the level priority of your choice to study Preventive medicine? | First  Second  Third  Not remember | | 1🗖🡪🡪  2🗖  3🗖  98🗖 | 🡪 C7 |
| B5 | | If Preventive medicine was not, what was your first choice? | General doctor  Dentistry  Traditional medicine  Nursing  Public Health  Medical technician  Not remember | | 1🗖  2🗖  3🗖  4🗖  5🗖  6🗖  98🗖 |  |
| B6 | | How did you feel when you had to choose the 2^nd^ choice? | Disappointed  Sad  Not so bad  I was accepted, no matter what specialty  Still satisfied because I was accepted by medical school  Not remember | | 1🗖  2🗖  3🗖  4🗖  5🗖  98🗖 |  |
| B7 | | What were the main reasons for your choice of Preventive Medicine? *(if more than one, then indicate their prioritizing by numbering them)* | **Characteristics of the specialty**  Privilege and advantages of a new profession  High salary or easy money-making  Travelling and meeting new people  There is less tension in the job  The high prestige of health jobs in general  **Personal preference**  Suitability to my personality and ability  One of my personal idol is a preventive doctor  Having an affinity for disease prevention  **Advance points**  Opportunity of scholarship for studying aboard  Lower entry criteria than other specialties  There is lower study burden than other specialties  It is easier to find a job if I study this specialty  **Influenced by others**  My idol is a preventive medical doctor  Family tradition  Family’s wish  Friends’ suggestion  Other (in detail)……………  Not remembered | | 1🗖  2🗖  3🗖  4🗖  5🗖  6🗖  7🗖  8🗖  9🗖  10🗖  11🗖  12🗖  13🗖  14🗖  15🗖  16🗖  97🗖  98🗖 | ***priority***  ***…***  ***…***  ***…*** |
| B8 | | Who had the biggest influence on your choice of Preventive Medicine? *(if more than one, then indicate their prioritizing by numbering them)* | Myself  Father/mother  Sister/brother  Relatives  Friend  Partner/wife/husband  Idolized medical doctor  Others (in detail) …………  Not remember | | 1🗖  2🗖  3🗖  4🗖  5🗖  6🗖  7🗖  97🗖  98🗖 | ***priority***  ***…***  ***…***  ***…*** |
| B9 | | Had you searched for information about Preventive Medicine discipline before applying to join the medical university? | Yes  No | | 1🗖  2 🗖🡪🡪 | 🡪 C11 |
| B10 | | By which media did you find information about Preventive medicine? | Public communication media  Father/ mother  Brother/sister  Relatives  Friends  High school teachers  Other medical students  University enrollment guidance  Others (in detail) ………….  Not remember | | 1🗖  2🗖  3🗖  4🗖  5🗖  6🗖  7🗖  8🗖  97🗖  98🗖 |  |
| B11 | | Have you ever wanted to change the discipline to follow? | Not yet, I’m only in the 1^st^ year  I want or wanted to change to another specialty  I want to change to other professions outside health care  Never | | 1🗖  2🗖  3🗖  4🗖 |  |
| B12 | | Has your attitude towards preventive medicine changed? | Yes  No | | 1🗖  2 🗖🡪🡪 | 🡪 D1 |
| B13 | | When did this change happen? | Y1  Y2  Y3  Y4  Y5  Y6  Not remember | | 1🗖  2🗖  3🗖  4🗖  5🗖  6🗖  98🗖 |  |
| B14 | | If applicable, list three reasons for liking PM more *(if more than one, then indicate their prioritizing by numbering them)* | Interesting syllabuses (plenty practicing)  Less pressure  Changes in society (e.g. job opportunities)  Suitability to my personality and wishes  Gaining better knowledge about the discipline  Positive feedback from senior students in the same discipline  The encouragement and advocating from lecturers  Others (in detail)…………… | | 1🗖  2🗖  3🗖  4🗖  5🗖  6🗖  7🗖  97🗖 | ***priority***  ***…***  ***…***  ***…*** |
| B15 | | If applicable, list maximally three reasons for disliking PM more *(if more than one, then indicate their prioritizing by numbering them)* | Inappropriate curriculum  Heavier study burden  Fewer job opportunities  Unsuitable to my personality and wishes  Yet to understand enough about the discipline  I see this profession less prestige than other medical specialties (e.g. general doctor)  Negative feedback from senior students  Negative feedback from lecturers  Others (in detail)…………… | | 1🗖  2🗖  3🗖  4🗖  5🗖  6🗖  7🗖  8🗖  97🗖 | ***priority***  ***…***  ***…***  ***…*** |
| **C** | | **Choosing future job** |  | |  |  |
| C1 | | What field would you prefer to work after graduation?  *(you can choose more than one option)* | Yet to decide  Preventive Medicine/Public Health  Clinical practice in hospital  Laboratory/fundamental sciences  Any specialty in hospital  Foreign Health organization  Outside of health system  Others (in detail)…………………… | | 1🗖  2🗖  3🗖  4🗖  5🗖  6🗖  7🗖  97🗖 |  |
| C2 | | If you want to work in a hospital, where would you prefer to work? | Don’t want to work in a hospital  Yet to decide  State hospitals  Provincial hospitals  Suburban hospitals  Commune hospitals  Foreign hospitals  Others (in detail)…………………… | | 1🗖  2🗖  3🗖  4🗖  5🗖  6🗖  7🗖  97🗖 |  |
| C3 | | Which factors will influence your choice of a future job *(if more than one, then indicate their prioritizing by numbering them)* | Suitable to my preference and ability  Within my specialty  Staying in big cities  High salary  Good working condition  Promotion opportunity  Chances for further study  Travelling abroad  Staying close to my family  Easy to find a job  Working for community in my hometown  Others (in detail)…………………… | | 1🗖  2🗖  3🗖  4🗖  5🗖  6🗖  7🗖  8🗖  9🗖  10🗖  11🗖  97🗖 | ***priority***  ***…***  ***…***  ***…*** |

**PLEASE CHECK TO ENSURE THAT YOU DO NOT MISS ANY QUESTION**

**THANK YOU VERY MUCH FOR YOUR TIME!**
